# Spatial and temporal distribution of infectious disease epidemics, disasters and other potential public health emergencies in the World Health Organisation Africa region, 2016–2018

**DOI:** 10.1186/s12992-019-0540-4

**Published:** 2020-01-15

**Authors:** Ambrose Otau TALISUNA, Emelda Aluoch OKIRO, Ali Ahmed YAHAYA, Mary STEPHEN, Boukare BONKOUNGOU, Emmanuel Onuche MUSA, Etienne Magloire MINKOULOU, Joseph OKEIBUNOR, Benido IMPOUMA, Haruna Mamoudou DJINGAREY, N’da Konan Michel YAO, Sakuya OKA, Zabulon YOTI, Ibrahima Socé FALL

**Affiliations:** 1 World Health Organization, Regional Office for Africa, Health Emergencies programme, Brazzaville, Congo; 2 Population Health Unit, Kenya Medical Research Institute - Wellcome Trust Research Programme, P.O. Box 43640-00100, Nairobi, Kenya; 3 World Health Organization, Emergency Response Department, Health Emergencies programme, Geneva, Switzerland

**Keywords:** African region, Disaster, Epidemic, Event, International health regulations, Public health emergency, Public health emergency of international concern, World Health Organisation

## Abstract

**Background:**

Emerging and re-emerging diseases with pandemic potential continue to challenge fragile health systems in Africa, creating enormous human and economic toll. To provide evidence for the investment case for public health emergency preparedness, we analysed the spatial and temporal distribution of epidemics, disasters and other potential public health emergencies in the WHO African region between 2016 and 2018.

**Methods:**

We abstracted data from several sources, including: the WHO African Region’s weekly bulletins on epidemics and emergencies, the WHO-Disease Outbreak News (DON) and the Emergency Events Database (EM-DAT) of the Centre for Research on the Epidemiology of Disasters (CRED). Other sources were: the Program for Monitoring Emerging Diseases (ProMED) and the Global Infectious Disease and Epidemiology Network (GIDEON). We included information on the time and location of the event, the number of cases and deaths and counter-checked the different data sources.

**Data analysis:**

We used bubble plots for temporal analysis and generated graphs and maps showing the frequency and distribution of each event. Based on the frequency of events, we categorised countries into three: Tier 1, 10 or more events, Tier 2, 5–9 events, and Tier 3, less than 5 or no event. Finally, we compared the event frequencies to a summary International Health Regulations (IHR) index generated from the IHR technical area scores of the 2018 annual reports.

**Results:**

Over 260 events were identified between 2016 and 2018. Forty-one countries (87%) had at least one epidemic between 2016 and 2018, and 21 of them (45%) had at least one epidemic annually. Twenty-two countries (47%) had disasters/humanitarian crises. Seven countries (the epicentres) experienced over 10 events and all of them had limited or developing IHR capacities. The top five causes of epidemics were: Cholera, Measles, Viral Haemorrhagic Diseases, Malaria and Meningitis.

**Conclusions:**

The frequent and widespread occurrence of epidemics and disasters in Africa is a clarion call for investing in preparedness. While strengthening preparedness should be guided by global frameworks, it is the responsibility of each government to finance country specific needs. We call upon all African countries to establish governance and predictable financing mechanisms for IHR implementation and to build resilient health systems everywhere.

## Background

There is an increasing recognition of the threat epidemics, disasters and public health emergencies pose to global health security and to the livelihoods of people, beyond their impact on human health. Each one of the 47 countries in the World Health Organization (WHO) African Region is at risk of health security threats [1, 2]. Emerging and re-emerging infectious diseases with a potential to spread internationally continue to challenge not only the fragile health systems in developing countries but also in the developed countries, creating enormous human and economic toll.

According to the WHO International Health Regulations-IHR (2005) “disease” means an illness or medical condition, irrespective of origin or source, that presents or could present significant harm to humans, while “event” means a manifestation of disease or an occurrence that creates a potential for disease [3]**. Epidemic** refers to an increase, often sudden, in the number of cases of a disease above what is normally expected in a given population in a given area. While “o**utbreak”** carries the same definition of epidemic, it is often used for a more limited geographic area. **Pandemic** refers to an epidemic that has spread over several countries or continents, usually affecting many people [4]. A disaster is a serious disruption of the functioning of a community or a society causing widespread human, material, economic or environmental losses which exceed the ability of the affected community or society to cope using its own resources [5]. It could also be defined as a situation or event, which overwhelms local capacity, necessitating national or international level external assistance [6]. A public health emergency on the other hand is the occurrence or imminent threat of an illness, event or health condition that poses a substantial risk of a significant number of human fatalities or incidents or permanent or long-term disability [7]. The declaration of a state of public health emergency permits a country or state to suspend certain regulations and change the functions of the country or state agencies. The most extreme is a public health emergency of international concern (PHEIC), which is an extraordinary event which is determined to constitute a public health risk to other States through the international spread of disease and to potentially require a coordinated international response [3].

Every year, the WHO African Region records more epidemics, disasters and potential public health emergencies than what is recorded in other WHO regions. A recent temporal analysis indicates that the risk of emerging and re-emerging infectious disease epidemics has risen [1, 8]. This could partly be due to better surveillance and improved reporting, but it could also be due to real events, including: the growth of cross-border movement and international travel, increasing human population density, rapid and unplanned urbanisation, recurrent political and other social conflicts and growth of informal settlements [9]. Other factors include the consequences of climate change-although the indirect effects of climate change are complex. Moreover, changes in the way humans and wild animals interact and changes in trade and livestock farming and inadequate IHR capacities are also contributing factors [9–12].

The recent devastating effects of the cyclone in the southern African region, the current Ebola virus disease (EVD) epidemic in the Democratic Republic of Congo (DRC), the 2013–2016 EVD epidemic in West Africa and the humanitarian crisis in Northern Nigeria, South Sudan, DRC, Central African Republic(CAR) and Mali are poignant reminders of the local and global threat of epidemics and other public health emergencies. The unprecedented spread of the West Africa EVD epidemic and its catastrophic consequences were attributed to several national and international weaknesses triggering a wake-up call to the global community [13, 14]. Beyond the obvious health impacts of epidemics are major socioeconomic impacts, devastation to livelihoods and the economies of the affected countries. For example, the EVD epidemic in West Africa – in Liberia, Guinea and Sierra Leone in 2013–2016 – had a huge negative impact on lives, livelihoods and communities and caused wider socioeconomic losses [15]. More than 28,000 people were infected and more than 11,000 of them died. The estimated economic loss in the most affected countries was approximately US$2.2 billion, or about 16% of their collective income [16–18].

To provide an evidence base for an investment case for public health emergency preparedness in Africa, we analysed the spatial and temporal distribution of epidemics, disasters and other potential public health emergencies in the WHO African Region for the period 2016 to 2018. This led to a comprehensive compilation of data on disease epidemics, disasters and other potential health emergencies and where they occurred. The information was then summarized by country and by year for the period 2016–2018.

## Methods

### Data sources

The key data sources used were: the WHO African Region’s Weekly bulletins on epidemics and other emergencies, and the WHO-Disease Outbreak News (DON), and the Emergency Events Database (EM-DAT) of the Centre for Research on the Epidemiology of Disasters (CRED). Additional sources included: the Program for Monitoring Emerging Diseases (ProMED) and the Global Infectious Diseases and Epidemiology Online Network (GIDEON). As part of the analysis, weekly data on epidemics and other emergencies were extracted from the Weekly bulletins compiled by the WHO Health Emergencies Programme from the website https://www.afro.who.int/health-topics/disease-outbreaks/outbreaks-and-other-emergencies-updates. Data on infectious disease epidemics, disasters and other emergencies were compiled from the reports published on the website from 2017 to 2018.

The WHO-DON database is operated in Geneva and is based on notifications of public health events caused by various infectious pathogens and other hazardous substances submitted to the WHO by member states as required under the IHR (2005). Public health events that are unusual or associated with increased risk to humans are published weekly in the DON website (https://www.who.int/csr/don/archive/year/2016/en/). Data on infectious disease epidemics and other health emergencies were compiled from the reports published on the website from 2016 to 2018. From these two sources, we were able to compile a database containing information on 194 epidemic and pandemic diseases and 47 other public health emergencies.

The EM-DAT epidemic data is compiled from sources such as the United Nations (UN) agencies, non-governmental organizations, insurance companies, research institutes and press agencies who report epidemic events. These data are available at the Office of Foreign Disaster Assistance(OFDA) of the United States or the CRED International Disaster Database and can be accessed through the EM-DAT website (www.emdat.be). Data were obtained through an official request and the data elements included were the actual location, origin and start and end dates of the epidemic/event. These databases were received via email on 28 January 2019. The EM- DAT database contained 41 epidemics/events for the period 2016 to 2018.

The ProMED database is an open source online reporting system established in 1994 by the Federation of American Scientists and the United Kingdom Space Agency-SATELLIFE, to provide up-to-date information on outbreaks of diseases and exposures to toxins that affect humans, as well as, animals and crops grown for food. The information disseminated by ProMED is collected through media reports, official reports, online summaries and by local observers, and later verified by a team of experts before it is posted on the ProMED website (www.promedmail.org) where it can be accessed freely. Data from ProMED contained 42 epidemics/events.

The GIDEON is a Global Infectious disease knowledge management tool that maintains up to date information on the latest trends in epidemiology and treatment. All the information available through GIDEON is from sources that are peer-reviewed and backed by scientific evidence. The information available through GIDEON is collected from various sources including ProMED. The information is accessed and collated through a system of computer macros which includes a monthly search of PubMed against a listing of all GIDEON key words, and titles / abstracts of interest are reviewed. All available national Health Ministry publications [print and electronic] are scanned, as are standard publications of WHO and the US Centres for Disease Prevention and Control (US CDC). Additionally, relevant peer-reviewed publications are continually examined for relevant articles. The GIDEON database contained 119 events over the period 2016 to 2018.

#### Assembly of the data for infectious disease epidemics and other public health emergencies

Most of the databases used for this analysis included information on disease, time and location of the epidemic, the number of cases and deaths. Epidemic data from each data source were counter-checked with data from other sources. The various databases were compared based on disease, location, start and end times, and magnitude in terms of number cases and number of deaths. Where one epidemic was reported from different data sources, the record from the data source that had reported the disease causing the epidemic, the exact location of the epidemic, the exact start and end dates of the epidemic and the actual number of cases were used, otherwise we defaulted to using WHO data. An epidemic or health emergency that started in one year and continued into consecutive years, was recorded as one event unless there was evidence of totally independent epidemiological occurrence. If an epidemic ran for more than one year, it was assigned to the year when the bulk of the epidemic occurred or to the midpoint of the epidemic if it ran beyond two years, or whichever was appropriate.

#### Data analysis

We used bubble plots to show temporal trends of all epidemics and disease specific epidemics due to Cholera, Measles, Meningitis and Viral Haemorrhagic Diseases (Crimean Congo, Dengue Fever, Ebola Virus Disease, Lassa Fever, Marburg Virus Disease, Rift Valley Fever, Yellow Fever and Viral Haemorrhagic Fevers of unknown or unspecified origin. We computed the frequencies of each event and generated maps showing the frequency and distribution of each event. We categorised the 47 countries into three tiers based on the frequency of events -Tier 1, 10 or more events, Tier 2, 5–9 events, and Tier 3, less than 5 or no event and then compared the frequency of events to the IHR summary index for all 13 IHR capacity technical areas based on data from the IHR State Party Annual report for 2018. Finally, we generated stacked frequency bar graphs of all the epidemic and other public health emergency events by country for the period 2016–2018.

## Results

**Fig. 1:**
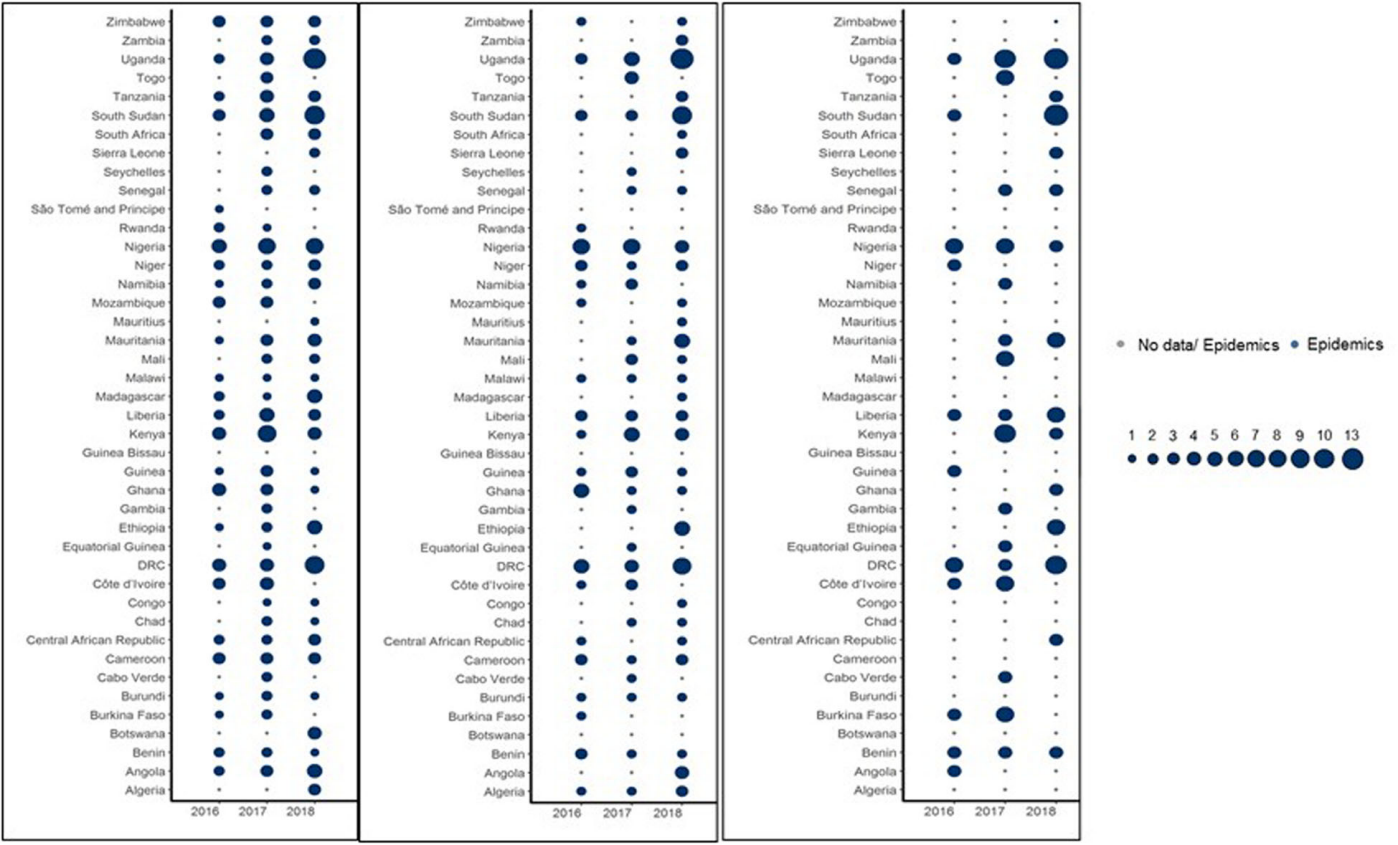
Bubble plots showing temporal trends of all epidemics from all diseases (Left Panel); Epidemics caused by Cholera, Measles, Meningitis and Viral Haemorrhagic Diseases (Crimean Congo, Dengue, Ebola, Lassa, Marburg, Rift Valley Fever, Yellow Fever and Viral Haemorrhagic Fever of unknown or unspecified origin) (Middle Panel); and epidemics caused by Viral Haemorrhagic Diseases (Right Panel) that occurred between 2016 and 2018 in the WHO African Region

Over 260 infectious disease epidemics, disasters and other potential public health emergencies were identified between 2016 and 2018 (Fig. 1). A public health event was reported in almost all countries in the WHO Africa Region, Cholera being the most geographically widespread. Ebola Virus Disease, Lassa Fever and Marburg Virus Disease were focal and mostly impacted specific areas in the region. Cholera and Measles caused outbreaks in many parts of the WHO African Region, while outbreaks of Meningitis and Crimean-Congo Haemorrhagic Fever primarily occurred in a few areas.

The top five causes of outbreaks during the period 2016–2018 were: Cholera, Measles and Viral Haemorrhagic Diseases, such as Ebola Virus Disease, Yellow Fever, Dengue Fever, Lassa Fever, and Rift Valley Fever. Other causes were Malaria and Meningitis.

**Fig. 2:**
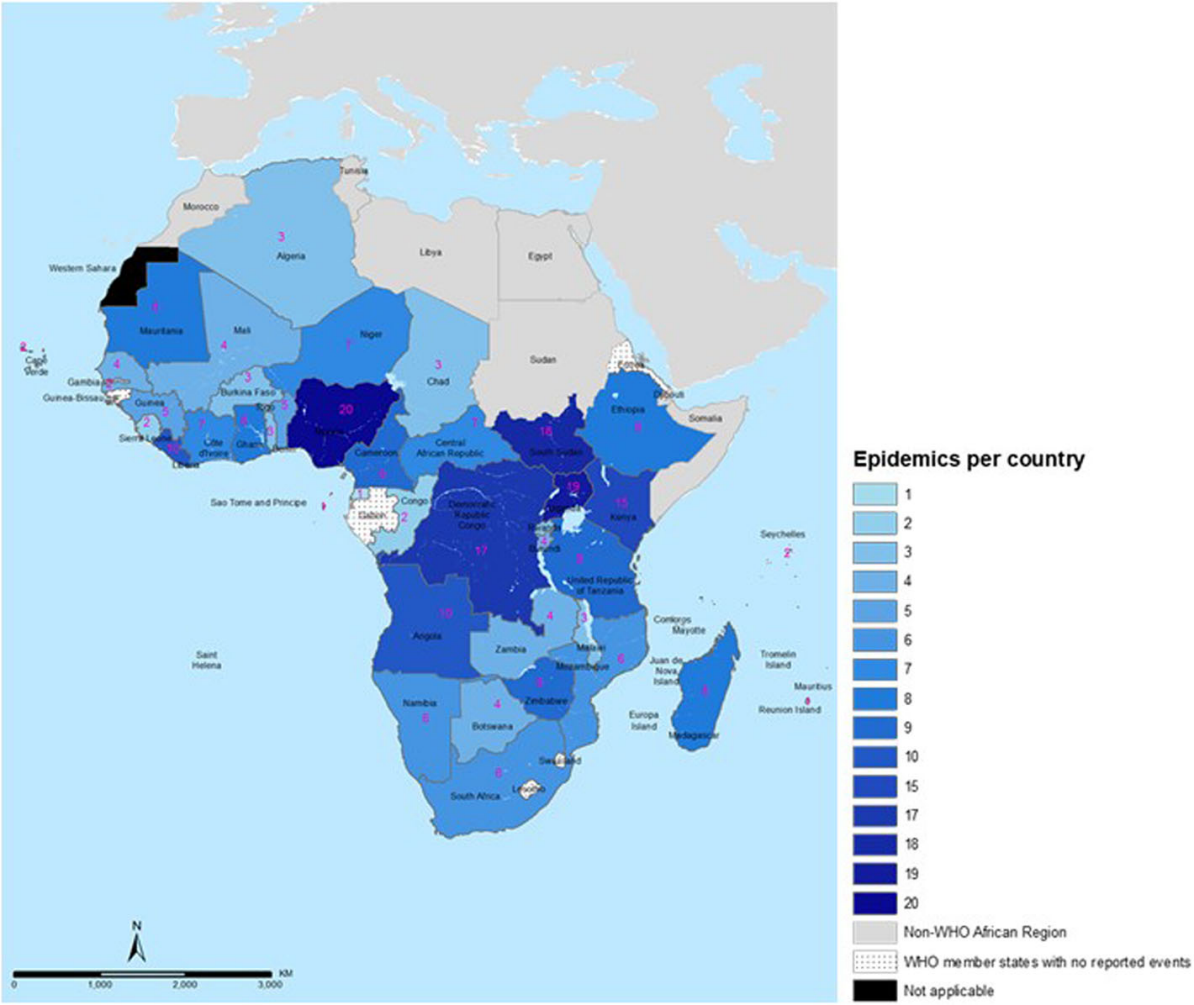
Map showing the number of epidemics and other public health emergencies in the WHO African region, 2016–2018

**Fig. 3:**
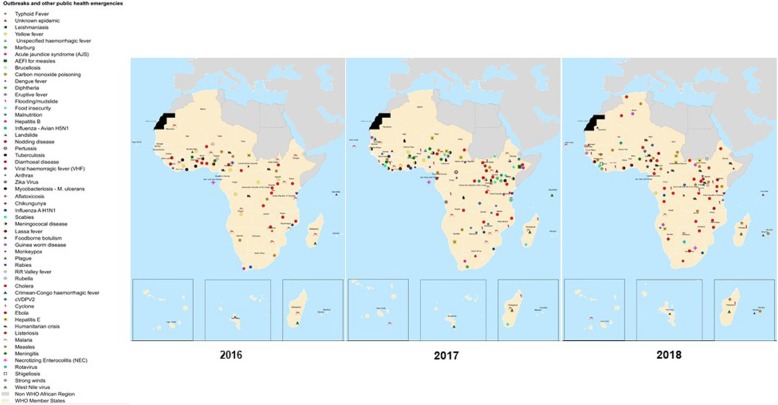
Map showing the spatial distribution of epidemics and other public health emergencies in the WHO African region, 2016–2018

**Fig. 4:**
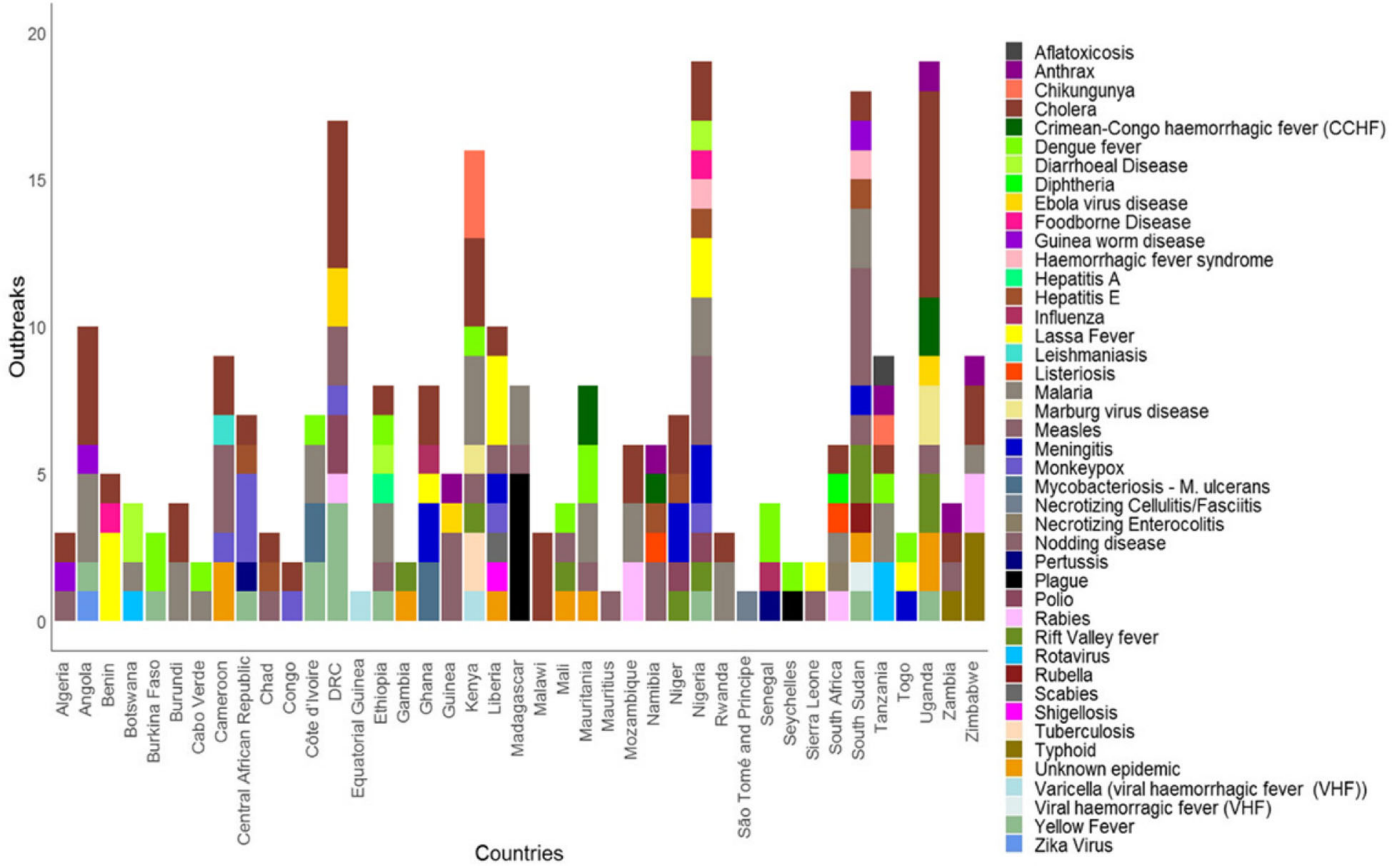
A stacked bar graph of all the epidemic events by disease in the countries of the WHO African region, 2016–2018

**Fig. 5:**
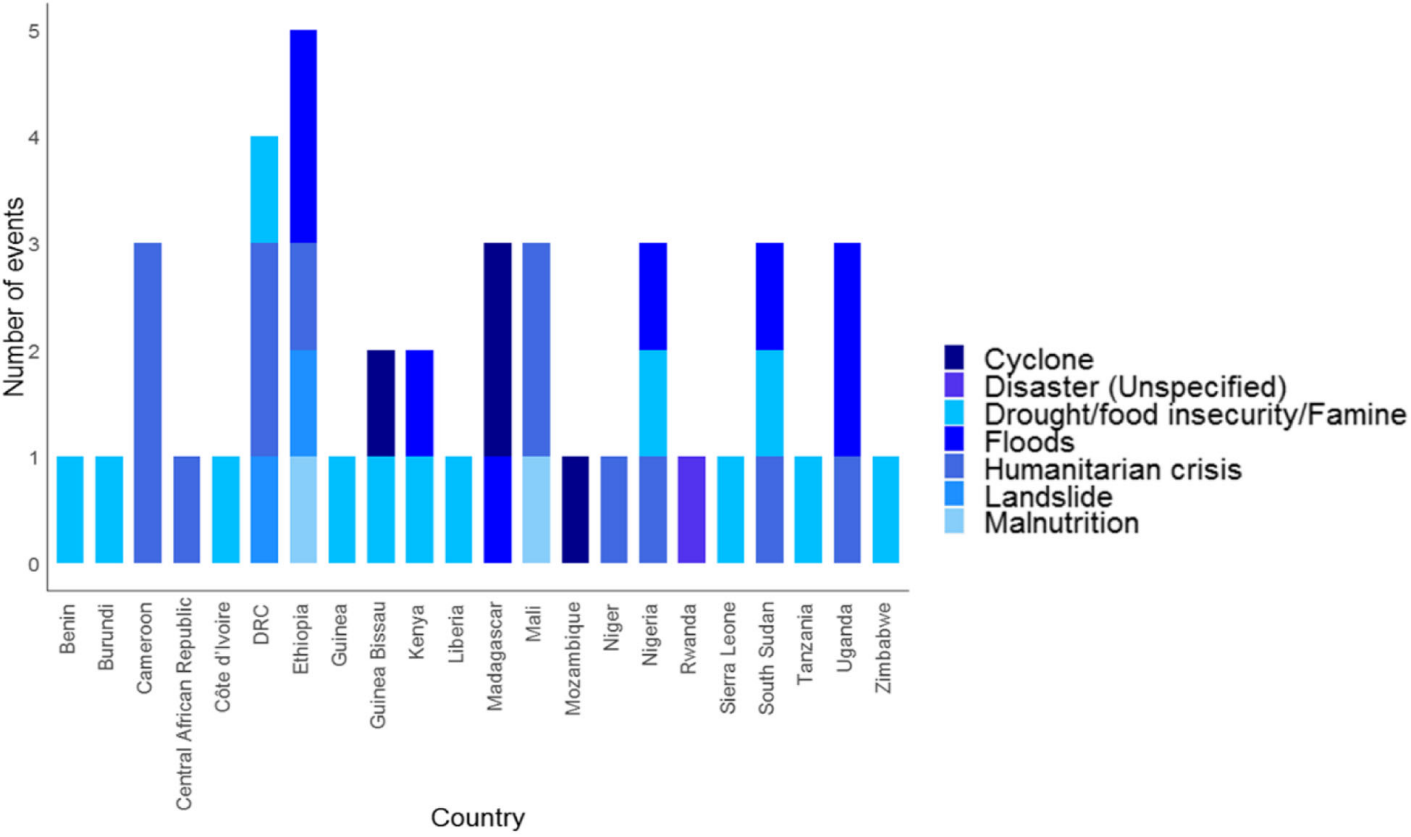
A stacked bar graph of all the other public health emergencies by countries of the WHO African region, 2016–2018

Figure 2 illustrates the frequency and spatial distribution, while Fig. 3 are maps showing the infectious disease-causing agent responsible for the epidemic or the public health emergencies in 2016, 2017 and 2018. Fig. 4 is a stacked chart of all the epidemics by disease for each country where an epidemic was reported, and Fig. 5 is a stacked chart of other public health emergencies by event that occurred during 2016–2018 in the countries of the WHO African region.

Forty-one countries (87%) had at least one epidemic during the period 2016–2018 and 21 of them (45%) had at least one epidemic per year (Fig. 1). These data demonstrate that infectious disease epidemics continue to emerge and re-emerge predictably in certain places and unpredictably in other places (Fig. 2 and Fig. 3). Further, 22 countries (47%) had other potential public health emergencies during the period 2016–2018, including disasters and humanitarian crises (Fig. 5). Five countries (the DRC, Kenya, Nigeria, South Sudan and Uganda) reported over 15 public health events during the period 2016–2018.

Most of the reported epidemics, disasters and other potential public health emergencies appear to have occurred in the same countries—a few countries have the highest number of epidemics and most of them are in the Central, West and the East African regions. These countries could be categorized as the epicentres of epidemics. The number of events reported was used to classify countries into three priority tiers based on the frequency of epidemics during the period 2016–2018. High priority (tier 1) are those countries that had 10 or more epidemics during the period 2016–2018. These countries also had limited or developing IHR capacities, based on the 2018 IHR annual reporting data (Table 1). Moderate priority (tier 2) are those countries that had 5–9 epidemics during the period 2016–2018. Low priority (tier 3) are those countries with fewer than 5 epidemics or had no epidemic recorded or reported through the tracking portals detailed in the methods section (Table 1).

**Table 1:**
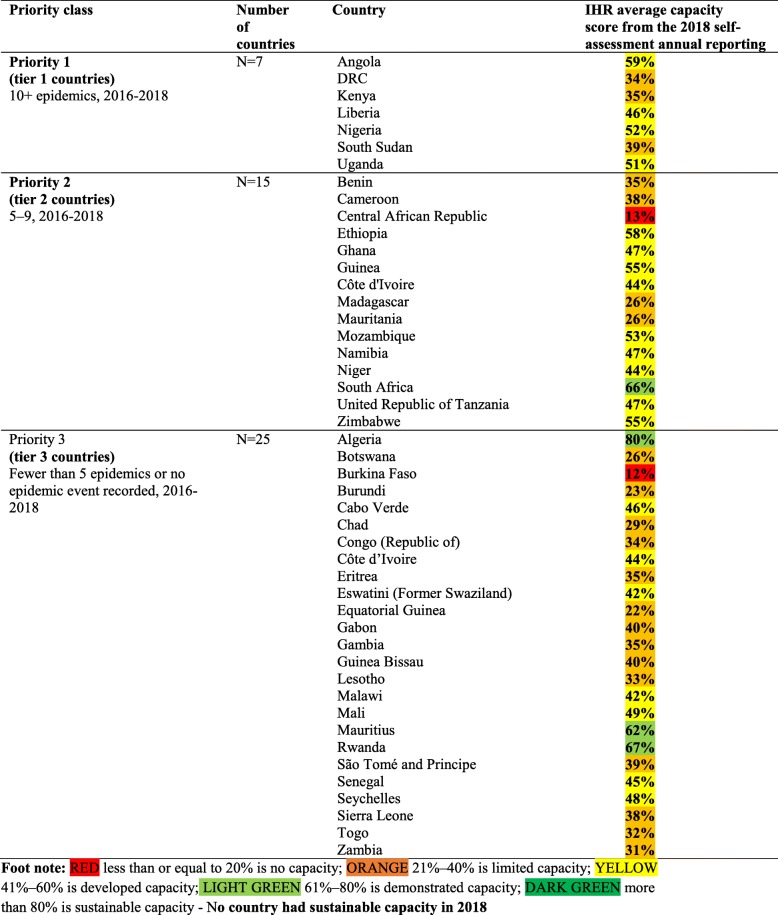
Prioritization of countries, based on frequency of infectious disease epidemics, 2016–2018

## Discussion

This analysis demonstrates that the entire WHO African region continues to be at risk of health security threats—a blunt reminder of the need for robust public health emergency preparedness and response systems everywhere. Further, our analysis suggests that there is an increase in the frequency of infectious disease epidemics, disasters and other potential public health emergencies in the WHO African region. This observation is similar to what was reported in a 2016 WHO African region review of the occurrence of epidemics for the period 1970–2016 [1]. There were fewer epidemics of Measles and Viral Haemorrhagic Diseases in the 2016 review compared to this review. However, Cholera was common in the 2016 review and is still very common in this review, while Polio and Malaria are less common in this review.

A caveat to this observation is that there has been improvement in public health surveillance, reporting and data collation practices on epidemics and better diagnostics to identify the disease-causing organisms, hence, these trends could be confounded by this bias. Nonetheless, the number of epidemics remains high—more than 100 epidemics were recorded in both 2017 and 2018, compared to 58 epidemics recorded in 2016. The increase in the frequency of epidemics and other public health emergencies is probably due to the combined impacts of rapid population growth resulting in increased population density and unplanned urbanization creating conditions that are conducive for the spread of diseases and leading to a greater scale of epidemics. Some of the changes in the infectious disease transmission patterns are likely a consequence of climate change, but the underlying causal relationships are also complex [9]. We know that vectors and disease-causing pathogens and hosts require optimal climatic conditions to survive and produce; chief among them are temperature and precipitation, which obviously are changing. Additionally, complicit are health system weaknesses which hamper early detection and prompt effective response.

Reforms in the WHO health emergency programme post the 2013–2016 West Africa Ebola are beginning to bear results. For example, the time taken to control outbreaks in the WHO African Region, has reduced from an average of 418 days (well over a year) in 2016 to 51 days (under two months) in 2018. Further, epidemics are being detected and responded to faster [19]. Moreover, African countries are commended for their willingness to assess and subsequently strengthen their IHR capacities to prepare for and respond to emergencies [20]. To date, forty-six countries in the WHO African region have had their IHR capacities assessed through joint external evaluation (JEE) [11, 18]. This is the highest number of JEEs among the WHO regions. Importantly, 30 of the countries that have conducted a JEE have subsequently developed their national action plans for health security (NAPHS) to address the identified gaps [18]. Nigeria and Uganda are two practical examples of countries showing the benefits of investing in building IHR capacities. Both countries have been able to detect and respond much faster to outbreaks. In Uganda, improvements in public health surveillance have been shown to substantially improve the response time for Viral Haemorrhagic Disease epidemics, thus reducing the scale and length of epidemics [21]. Similarly, Nigeria is strengthening implementation of the IHR. In 2016, the Nigeria Centre for Disease Control (NCDC) was established and has been instrumental in improving coordination of public health emergency preparedness and response. This was demonstrated during the 2017 Monkey pox outbreak. Previously, Monkey pox surveillance was hindered by inadequate actionable data as Monkey pox was not a notifiable disease. A more sensitive surveillance system led to the detection of the index case in 2017 after 39 years of the last recorded case [22]**. S**urveillance for the early detection of viral spillovers and advanced genetic characterization for determination of the origin of the outbreak was improved through collaboration with partners [23–25]. The response of the NCDC in creating awareness and providing tools for surveillance led to the detection of more suspected monkey pox cases in 21 states. In addition, increased coordination and partnerships from the Federal Government, the States and other collaborating partners, led to its containment, largely credited to strengthened surveillance and laboratory diagnosis support.

There are several worrying trends. First, there is evidence that Viral Haemorrhagic Disease epidemics that were previously rare have recently caused devastating epidemics in the region [26, 27]. For instance, Ebola and Marburg Virus Diseases (MVD), previously known to be rare, have recently caused major epidemics in Liberia, Guinea and Sierra Leone (2013–2016) [28–31], in Uganda (2017 and 2018) [32, 33], and in the Democratic Republic of the Congo (2018 and 2019) [34–37]. A 2016 comprehensive regional risk assessment and mapping for all epidemics reported in Africa between 1970 and 2016 revealed that the epicentres for the Ebola virus disease and Marburg virus disease epidemics were mainly in East and Central Africa, except for the West Africa Ebola virus disease epidemic [1]). Second, several epidemics of meningococcal meningitis recently occurred outside the meningitis belt, showing a high likelihood that the areas at risk are expanding [2]. Third, Cholera is transitioning from an epidemic to an endemic disease. In 2017, over 150,000 Cholera cases, including over 3000 deaths were reported from 17 countries in the African region and countries, such as Chad and Zambia, where Cholera epidemics were rare prior to 2016, reported epidemics [38]. Fourth, many of the countries at risk of epidemics also continue to suffer from disasters and humanitarian crises that disrupt livelihoods and the economy of the affected countries, thus constituting a dual threat.

We explored the dual occurrence of infectious disease epidemics, disasters and humanitarian crises. A comparison of data on infectious disease epidemics, disasters and other potential public health emergencies suggest that certain countries have a high frequency of infectious disease epidemics, as well as, a high frequency of other public health emergencies including: drought, flooding, cyclones and humanitarian crisis due to conflict. From the data assembled it is evident that several countries in tier 1 such as the Democratic Republic of the Congo (DRC), Kenya, Uganda, South Sudan also witnessed more humanitarian crisis events than other countries during the period 2016–2018. There are several factors that could explain these observations. Firstly, most of these epicentres are in the tropical savannah or tropical rain forests, which are ecologically prone to numerous infectious diseases. Secondly, most of these countries have had recurrent civil, social and political strife, which has often resulted into refugee and internally displaced populations, living in overcrowded settings with poor living conditions, inadequate access to clean water, food, shelter, health and other social services. Thirdly, most of them have inadequate health systems with poor access to quality health services. Fourthly, several of them have had challenges with governance and leadership and consequently limited political and financial commitment for building resilient health systems. The latter, together with lessons from recent disasters in southern Africa highlight the negative compounding effects of humanitarian crisis and infectious disease epidemics, suggesting obvious benefits of cross linkages and the potential value of leveraging financing for humanitarian crises to build IHR capacity [39].

It is increasingly apparent that emergency preparedness and response is best achieved within strong health systems for universal health coverage(UHC) [40]. In countries with weak health systems, response to health emergencies is slow or inadequate, and outcomes are poorer. Indeed, the JEE index scores correlate with various health metrics, including: life expectancy, under-five year old mortality, disability-adjusted life years, and other measures of social and economic development [41]. Consequently, efforts to strengthen health security should be integrated within health system strengthening. This would prevent duplication of efforts and wastage. However, how best to achieve or measure this integration is not clearly defined [40, 42]. The ability of deadly pathogens to exploit weak health systems was clearly demonstrated in the Ebola outbreak of 2013–2016 [13]. However, there is paucity of data on the benefits of integrating health security with health system strengthening. Several components are cross cutting and improvements or deficiencies in one, often impact the other. Nevertheless, evidence on the public health, economic, social and political dividends to support the integration of these two approaches is urgently needed and would be insightful to policy makers and funders. We are investigating the potential dividends of investing in an integrated approach to UHC and health security.

All countries in the WHO African region have embraced the revised IHR monitoring and evaluation framework [20], including: mandatory annual reporting, JEEs, after action reviews (AARs) and simulations and exercises (SIMEX). Most of the countries have subsequently developed and costed their NAPHS. However, the same cannot be said about mobilizing the financing for preparedness. Based on the 30 costed NAPHS, the financial needs for health security over next 3–5 years vary from approximately USD 7 million in Eswatini to USD 700 million in the Democratic Republic of the Congo (DRC). The region-wide estimate is approximately USD 7–8 billion or approximately USD 2.5–3 billion annually, which translates to USD 2.5–3 per capita per annum [11, 18]. Most of the countries with the lowest JEE scores are either low or middle-income countries and will require substantial development assistance to fill the gaps identified in the JEEs. A September 2019 report commissioned and prepared by the World Bank Group for the Global Preparedness Monitoring Board on pandemic preparedness financing has suggested six broad areas namely: Mobilizing domestic resources; Mobilizing development assistance; Incentivizing countries to prioritize allocation of funds for preparedness; Leveraging the insurance model, accessing existing loans, grants and new funding; Mobilizing funding for research and development (R and D) and strengthening clinical research capacities; and finally sustainable financing to strengthen WHO’s capacity [43]. WHO needs to be better resourced to galvanize intergovernmental and non-state actors to finance health security preparedness. More importantly, there is a need for a global financing mechanism to support countries that are not likely to have the required resources to implement the IHRs fully. In the early 2000s, HIV/AIDS, Tuberculosis and Malaria were affecting millions of vulnerable populations. In 2002, the Global Fund was established as a partnership to accelerate the end of the “epidemics” of AIDS, Tuberculosis and Malaria. The Global Fund mobilizes and invests more than US$4 billion a year to support programs in more than 100 countries [44]. Public health emergency preparedness financing in the low and middle-income countries, requires similar bold partnerships with governments, civil society, technical agencies, bilateral and multilateral funding agencies, the private sector and the communities to surmount the challenging barriers, while embracing innovation. Importantly, there is an urgent need to conduct a landscape analysis of existing funding to leverage on the opportunities provided by funding for vertical disease programmes, climate change, the Global Fund, and private sector contributions. Further, there is a need for further reprioritization of the country plans. Moreover, countries need to explore opportunities for: sharing capacities through regional centres of excellence that are serviced by several member states; and setting up regional public health emergency response workforces. Finally, there is a need to explore additionality and counterpart financing mechanisms to incentivise countries that increase their domestic financing for preparedness.

Several of the major events reported here were cross border events or crossed from one country to another, which necessitated cross border collaboration to contain them. Over the last two decades WHO has supported several high level ministerial meetings on cross-border health issues. These ministerial level meetings led to the signing of protocols of cooperation and memoranda of understanding to facilitate containment of cross border public health challenges and to harmonize strategies and policies across countries. This led to improved operational coordination, improved epidemic response and synchronization of public health interventions for health systems strengthening, implementation of integrated disease surveillance and response (IDSR) and IHR. However, the institutionalisation of mechanisms and processes for cross-border information sharing and collaboration to effectively address epidemic preparedness remain challenging. For example, few countries have established mechanisms and systems for joint planning, joint implementation and joint monitoring and evaluation of strategies for prevention, preparedness, early detection and response to cross-borders events. Moving forward, countries need to identify the major challenges to cross border coordination, collaboration and operations for preparedness and response activities. This should be followed by identification of opportunities for strengthening cross border collaboration to address the identified issues and challenges, using the existing protocols, strategies and frameworks or formulating new ones. Finally, countries should establish effective and sustainable cross border collaboration mechanisms and formulate strategic actions, including inter country agreements for cross border health service delivery.

## Conclusions

The frequent and widespread occurrence of epidemics, disasters and other public health emergencies in Africa requires substantial investments in preparedness capacity in all countries. While investing in preparedness should be guided by global frameworks such as the IHR, it is the responsibility of each government to finance country specific needs. The ongoing momentum by countries and several stakeholders in implementing the IHR monitoring and evaluation framework should be followed by strong resource mobilization for the implementation of the NAPHS with a focus on prioritization within priorities. We call upon all African governments to establish governance and predictable sustainable financing mechanisms to accelerate IHR implementation and to build resilient health systems everywhere.

## Data Availability

The datasets generated and/or analyzed during the current study are available in the public domain as stipulated in the methods section of this paper.

## Abbreviations

CDC: Centres for Disease Prevention and Control (United States)
CRED: Centre for Research on the Epidemiology of Disasters
DFID: Department for International Development (United Kingdom)
DON: Disease Outbreak News
DRC: Democratic Republic of the Congo
EM-DAT: Emergency Events Database
EVD: Ebola Virus Disease
GIDEON: Global Infectious Disease and Epidemiology Online Network
IDSR: Integrated Disease Surveillance and Response
IHR NFP: International Health Regulations National Focal Point
IHR: International Health Regulations
IHRMEF: International Health Regulations Monitoring and Evaluation Framework
JEE: Joint External Evaluation
NAPHS: National Action Plan for Health Security
OFDA: Office of Foreign Disaster Assistance (United States)
PHE: Public Health Event
PHEOC: Public Health Emergency Operation Centre
PROMED: Program for Monitoring Emerging Diseases
SATELLIFE: SatelLife (United Kingdom Space Agency)
TDDAP: Tackling Deadly Diseases in Africa Programme (United Kingdom)
VHDs: Viral Haemorrhagic Diseases
WHO: World Health Organisation
